# Study design considerations in clinical trials testing transcutaneous stimulation for spinal cord injury

**DOI:** 10.1038/s41393-026-01190-5

**Published:** 2026-03-06

**Authors:** James Guest, Chet Moritz

**Affiliations:** 1 Neurological Surgery and the Miami Project to Cure Paralysis, The University of Miami Miller School of Medicine, Miami, FL USA; 2 Electrical and Computer Engineering, Rehabilitation Medicine, and Neurobiology and Biophysics, Center for Neurotechnology, and Amplifying Movement and Performance Lab, The University of Washington, Seattle, WA USA

**Keywords:** Medical research, Clinical trial design

## Abstract

**Study Design:**

Methodological review and expert perspective.

**Objectives:**

To examine the methodological challenges in designing rigorous clinical trials for transcutaneous spinal cord stimulation (tSCS) in chronic spinal cord injury (SCI), with particular focus on challenges of sham control implementation, and to propose alternative trial design approaches that balance scientific rigor with practical feasibility and ethical considerations.

**Setting:**

United States.

**Methods:**

We analyzed the design considerations that influenced the Up-LIFT pivotal trial, examining three critical constraints: the technical limitations of creating safe and convincing sham stimulation for extended protocols; the participant burden associated with traditional sham-controlled designs; and the heightened risks during the COVID-19 pandemic. We reviewed existing literature on placebo effects in neuromodulation, technical challenges of sham tSCS implementation, and ethical considerations specific to the SCI population. Alternative methodological approaches were evaluated, including sequential self-controlled designs, biomarker-guided approaches, and adaptive trial designs.

**Results:**

Traditional sham controls for tSCS face serious technical challenges because participants readily detect stimulation parameters, minimal currents produce detectable neuromodulatory effects, and extended protocols amplify these issues through knowledge sharing and functional feedback. Ethical concerns include substantial participant burden, potential for lessebo effects when a sham is suspected, and erosion of therapeutic relationships through prolonged deception. The COVID-19 pandemic added critical safety considerations for the vulnerable SCI population. Alternative designs, such as sequential self-controlled approaches, as implemented in Up-LIFT, can maintain scientific validity while addressing these constraints.

**Conclusion:**

The unique challenges of tSCS clinical trials necessitate innovative methodological approaches beyond traditional placebo-controlled designs. Sequential self-controlled designs, biomarker-guided studies, and adaptive trial methodologies offer scientifically sound alternatives that respect participant welfare while generating robust evidence. Future research should pursue dual paths: developing improved sham paradigms while advancing alternative trial methodologies suitable for neuromodulation-enhanced rehabilitation interventions.

## Introduction

### Background and significance of tSCS for SCI

Spinal cord injury (SCI) creates lifelong disability that has historically been considered permanent during the chronic phase [1]. However, recent advances in neuromodulation-enhanced rehabilitation [2], particularly transcutaneous spinal cord stimulation (tSCS), challenge this concept [3–5]. The non-invasive nature of tSCS makes it an especially promising intervention, as demonstrated by the recent Up-LIFT pivotal trial and subsequent FDA approval [6]. This milestone establishes an important precedent for innovation in neuromodulation therapy within the historically challenging SCI field.

The development of rigorous clinical trials for neuromodulation presents unique methodological challenges. Primary among these challenges is the difficulty of implementing adequate control conditions that can account for potential placebo effects while maintaining scientific validity and ethical standards [7]. This challenge is particularly prominent in trials combining tSCS with prolonged intensive rehabilitation, where participant engagement and continuous motivation are crucial and inextricable determinants of outcomes. The physical sensations associated with stimulation, the extended duration of treatment protocols, a sense of progress, interactions with therapists and scientists, and the close-knit nature of the SCI community combine to complicate the implementation of traditional sham controls.

### The Up-LIFT trial: Overview and design rationale

The design of the Up-LIFT trial has sparked important discussions about balancing scientific rigor with practical and ethical considerations in SCI neuromodulation clinical research [8]. This paper examines the key challenges in designing sham controls for tSCS trials, with a particular focus on the following:

1. Technical requirements for valid sham stimulation in extended treatment protocols.
2. Potential negative effects of deceptive sham conditions in prolonged rehabilitation trials.
3. Practical constraints imposed by the burden on participants and recruitment feasibility.
4. Impact of the COVID-19 pandemic on the Up-LIFT trial design.

### Scope and objectives

This analysis aims to inform future trial designs, discuss the complexity of placebo responses, and stimulate discussion about optimal approaches to demonstrating efficacy in SCI neuromodulation interventions. While randomized placebo-controlled trials (RCTs) remain the gold standard, we argue that alternative designs may be more suitable for specific SCI neuromodulation studies, particularly those involving extended rehabilitation interventions.

Although this paper focuses on tSCS, many challenges described—particularly sham feasibility, placebo/lessebo dynamics, and participant burden—are shared by other neuromodulation approaches, including epidural stimulation, vagus nerve stimulation, and non-invasive brain stimulation (e.g., TMS, tDCS) [9, 10]. Lessons from tSCS may thus inform trial design across neuromodulation domains.

## Therapeutic foundations of tSCS rehabilitation

### The essential interaction between stimulation and task practice

The therapeutic benefits of tSCS in chronic cervical SCI arise from a complex interaction between direct neuromodulatory effects, activity-dependent plasticity, and impacts on muscle function. The Up-LIFT study investigated these interactions by assessing sequential improvements in upper extremity function during an initial two-month phase focused exclusively on task practice, followed by a two-month phase that integrated tSCS with intensive task practice. Current evidence suggests that both components—neuromodulation and task-specific practice—are crucial for significant functional gains [11]. The stimulation is hypothesized to enhance the excitability of spinal circuits, enabling volitional control of movement and thus greater participation in rehabilitation, creating a permissive state for plasticity [12]. At the same time, concurrent task practice provides the specific voluntary activation patterns necessary to strengthen relevant neural pathways. Therefore, stimulation alone is unlikely to yield significant effects without concurrent task practice [13].

The dual effects of stimulation and task practice align with the contemporary understanding of neuroplasticity, where pre- and post-synaptic activity must coincide to promote lasting plasticity [14, 15]. Transcutaneous spinal stimulation makes post-synaptic activity in the spinal cord more likely to occur in response to descending inputs, providing the cause-and-effect neural activity needed to promote long-term Hebbian plasticity [16, 17].

Spontaneous post-SCI neurological recovery typically plateaus around one year after injury [1]. In studies on chronic stroke and SCI recovery, conventional and even intensive task practice rehabilitation typically reaches a functional plateau beyond which additional recovery is difficult to achieve [1, 18–21]. The Up-LIFT trial design leveraged this knowledge by using participants as their own controls, anticipating that the recovery rate would be accelerated when task practice was combined with tSCS. Thus, the study was a prospective single-arm, sequential block treatment, multi-center, open-label study. A sham arm was not utilized, although it was carefully considered, as detailed in section 4 below.

### Implications for control condition design

The therapeutic understanding of how tSCS facilitates recovery has direct implications for control condition design in clinical trials.

#### Engagement and expectation effects

Inadequate sham stimulation may be detrimental because participants who perceive insufficient or ineffective stimulation could experience decreased motivation, altered effort levels, and diminished engagement—factors that directly impact rehabilitation outcomes independent of the stimulation’s physiological effects.

#### Protocol duration constraints

The prolonged treatment period necessary to achieve significant improvements due to the combination of rehabilitation and stimulation [5] makes traditional crossover designs particularly challenging. Such extended protocols increase the risk of participant dropout and elevate the likelihood of sham detection over time.

#### Interdependent components

The essential interaction between stimulation and task practice creates a complex intervention in which the components are not merely additive. This interdependence complicates the interpretation of isolated control conditions that separate these components and may underestimate the synergistic effects.

These considerations directly informed the Up-LIFT trial’s approach to controlling for non-specific effects while maintaining scientific rigor and feasibility. By using each participant as their own control through a substantial rehabilitation-only run-in phase, the study addressed the confounding factors of individual variability while allowing for the assessment of the combined intervention effect. Importantly, all outcome measure testing in Up-LIFT was conducted without stimulation, examining the prolonged functional impact of stimulation combined with rehabilitation rather than temporary facilitation (i.e., neuroprosthetic) effects post-stimulation. Thus, the probability of a placebo effect due to stimulation was reduced.

## Placebo effects in neuromodulation-enhanced rehabilitation studies

### The spectrum of expectation and experience effects in tSCS trials

The complexity of placebo effects in tSCS trials extends beyond traditional considerations in therapeutic research, especially when neuromodulation is combined with intensive rehabilitation. The placebo effect - a measurable improvement in health or behavior not attributable to the treatment itself - is a well-documented phenomenon driven by expectations, beliefs, and neurobiological mechanisms, including dopamine and endorphin signaling [22]. Studies have shown that these effects can be substantial in certain neuromodulation applications [23], making their understanding in different contexts crucial for effective trial design and outcome interpretation. The largest reported placebo effects occur in conditions that have subjective outcomes, such as the reporting of changes in pain [24] or depression [25].

However, placebo-related phenomena exist on a spectrum from positive (placebo) to negative (lessebo and nocebo) expectations, producing opposite effects on efficacy outcome measures. While positive placebo effects can enhance outcomes in control groups, “lessebo” effects can diminish the magnitude of therapeutic effects in active treatment groups when participants are uncertain about receiving active therapy [26].

- ***Placebo effects****:* Positive expectations leading to improved outcomes despite receiving inactive treatment.
- ***Lessebo effects****:* Reduced benefits in active treatment groups due to uncertainty about receiving the actual intervention.
- ***Nocebo effects****:* Negative expectations leading to adverse outcomes or reduced benefits [27].

The same neurobiological systems associated with positive placebo responses may contribute to negative responses during lessebo and nocebo effects [23]. This was demonstrated experimentally, as nocebo effects on corticospinal excitability were observed when subjects were told that TENS would reduce their muscle force output [28].

**Fig. 1:**
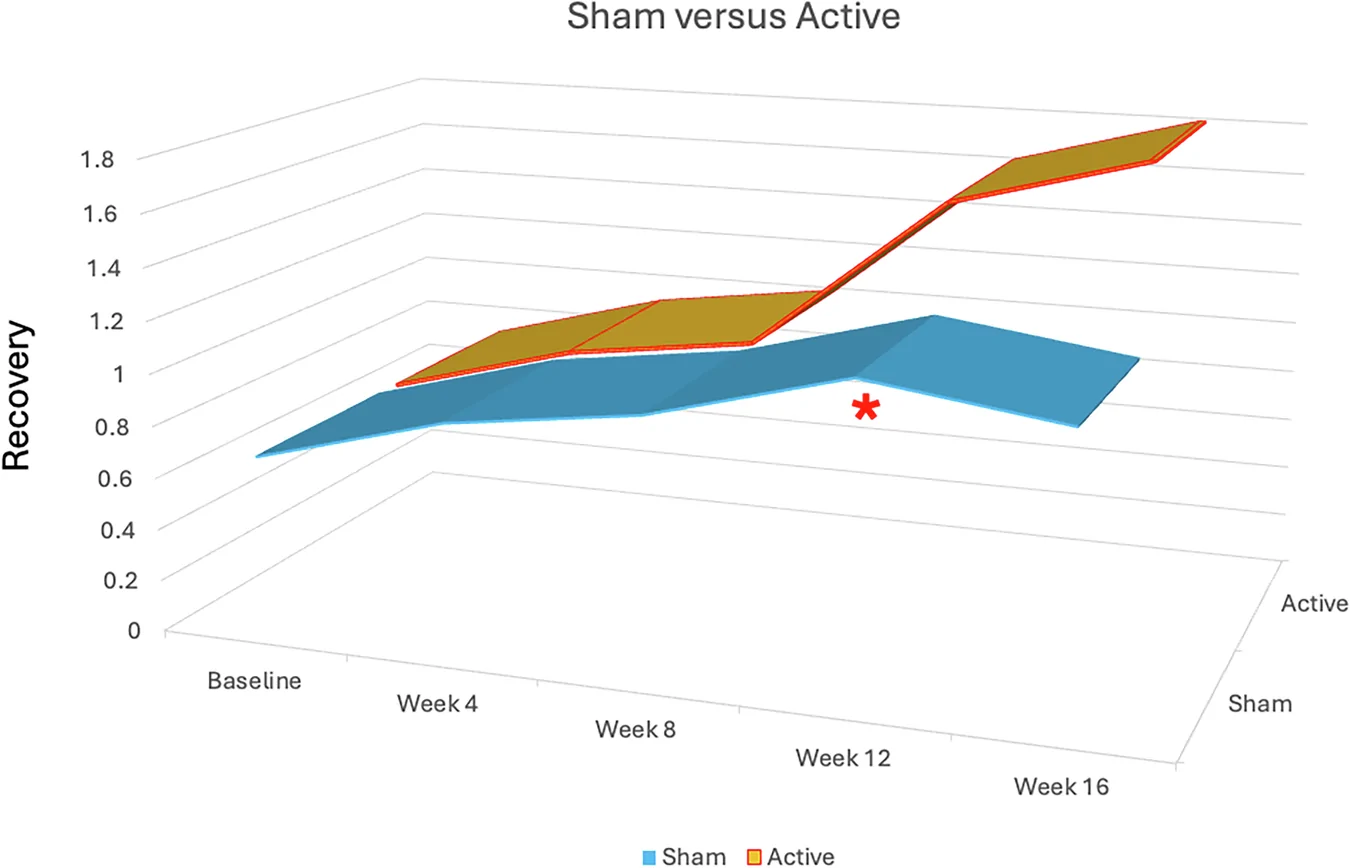
Potential Placebo and Lessebo Effects in a tSCS Clinical Trial. Illustrated is a hypothetical 16-week clinical trial with a task practice phase of 8 weeks, followed by randomization into a sham and active stimulation arms. Initially, the sham is effective, and a placebo effect is noted; however, after multiple sessions, the sham is no longer convincing (red asterisk), and the subjects are demotivated, resulting in a decrease in recovery.

Thus, expectation-driven responses create complex challenges for clinical trials in neuromodulation by potentially undermining the theoretical equipoise between task rehabilitation alone and its combination with active transcutaneous stimulation (Fig. 1). The psychological variables introduced can independently affect outcomes in both arms of a randomized study, complicating the interpretation of results. Moreover, many neuromodulation studies reporting large placebo effects focus on the *remission* of symptoms such as depression and pain [29], rather than on the acquisition of new motor or sensory function.

### Factors contributing to variable placebo effects in SCI stimulation studies

While placebo controls are fundamental to therapeutic research, their application in intensive rehabilitation trials for SCI presents unique methodological and ethical challenges. Interestingly, the literature shows inconsistent placebo responses in spinal cord stimulation studies. The magnitude of sham stimulation placebo effects has been minimal or absent in several studies, while being detectable in others [30–34]. This variability further complicates the design of appropriate control conditions for tSCS trials.

Placebo effects in neuromodulation involve these multiple interacting mechanisms [35]:

- **Psychological factors**: Expectations, conditioning, anxiety reduction, and belief/disbelief in treatment efficacy.
- **Neurobiological changes**: Release of endogenous opioids, dopamine, and changes in brain activity patterns.
- **Behavioral adaptations**: Altered engagement, attention, effort level, and adherence to rehabilitation protocols.
- **Social dynamics**: Quality of participant-provider interactions, perceived empathy, and communication.
- **Contextual factors**: Setting characteristics, device sophistication, and overall rehabilitation environment.

### Special placebo considerations in SCI rehabilitation trials

The SCI rehabilitation context presents distinct challenges for understanding and controlling placebo effects that go beyond those encountered in typical pharmacological trials. The effectiveness of combined task practice and tSCS paradigms critically depends on strong participant motivation and sustained engagement [36]—factors that are directly influenced by expectation and belief.

In this context, placebo and lessebo effects take on additional complexity for several reasons:

1. **Motivation as a therapeutic component**: Unlike medication trials, where taking a pill requires minimal effort, the success of rehabilitation depends on active and effortful participation. Awareness or suspicion of receiving sham stimulation could significantly reduce motivation, directly limiting functional improvements and potentially invalidating comparisons between control and treatment groups [37].
2. **Compounding effects over time:** The prolonged duration of rehabilitation trials (typically 8–24 weeks) implies that small initial differences in motivation or engagement can magnify over time. A participant who suspects they are receiving sham stimulation may gradually diminish their effort during sessions, leading to a self-fulfilling prophecy of reduced improvement.
3. **Trust and therapeutic alliance**: The relationship between participants and the research or clinical team is crucial for rehabilitation outcomes. A loss of trust due to perceived deception regarding treatment status can harm this therapeutic alliance, with consequences that extend beyond the immediate study context.
4. **Community dynamics:** The close-knit nature of the SCI community means that experiences are often shared among participants, potentially “unblinding” others and affecting their expectations and engagement.
5. **Ethical implications of effort requirements**: Asking participants with SCI to dedicate significant time and effort to intensive rehabilitation while potentially receiving non-therapeutic sham stimulation raises unique ethical considerations about the burden-to-benefit ratio in these research studies.

These considerations suggest that in lengthy tSCS rehabilitation trials, implementing a sham control may fundamentally alter the intervention being studied in ways that compromise both scientific validity and participant welfare. This creates a tension between methodological rigor as traditionally defined and meaningful clinical research in the SCI rehabilitation context.

### Variability and generalizability challenges

Beyond the general complexities of placebo effects in rehabilitation, the high degree of individual variability among people with SCI poses significant challenges for traditional randomized controlled trial designs. Matching groups at baseline is challenging. This heterogeneity encompasses multiple dimensions that can influence both treatment and placebo responses:

#### Sources of individual variability

- **Injury-specific factors**: Level, completeness, and time since injury create fundamentally different baseline conditions.
- **Psychological profiles**: Varying degrees of expectancy, motivation, and resilience directly impact the rehabilitation process.
- **Treatment history**: Prior exposure to rehabilitation approaches, pharmacotherapy, and neuromodulation technologies shapes expectations and responses.
- **Therapeutic relationships**: The quality of interactions with specific therapists and researchers varies between participants and sites.
- **Concurrent interventions**: Medications, physical therapy approaches, and assistive technologies differ between individuals.
- **Health status and genetics**: Comorbidities, secondary complications, polymorphisms [38], and overall wellness create different physiological contexts.
- **Support systems**: Family involvement, caregiver resources, and social factors influence adherence and outcomes.

This extensive variability has several critical implications for clinical trial design in tSCS research as described below.

### Implications for research design

A key pitfall in multimodal rehabilitation efficacy research is assuming that a single strategy, such as using placebos or random assignment, can resolve all threats to validity. While randomization and placebos may control some confounds, they do not address every source of bias [39].

1. **Sequential designs may offer advantages**. Within-subject comparisons can better control for individual variability than between-group designs, supporting the approach used in the Up-LIFT trial, where participants served as their own controls. It is also important to note that the trial design allowed for parameter optimization on the individual level [40]. Statistical comparisons of outcomes following each treatment phase allowed for recovery progress to be tracked longitudinally as the treatment paradigm changed.
2. **Sample size challenges**: The heterogeneity of the SCI population means that larger sample sizes would be needed for traditional RCTs to achieve sufficient statistical power—a particular challenge given the relatively small SCI population. Within-subject designs can provide greater statistical power [41].
3. **Subgroup responses**: Treatment effects may exist only in specific subgroups of participants, which can be obscured in analyses of overall group means.
4. **Responder analyses**: Identifying factors that predict positive responses to tSCS may be more clinically valuable than simple group comparisons [6].
5. **Mixed methods approaches**: Integrating qualitative data about participant experiences with quantitative outcomes may provide more comprehensive insights.

These challenges suggest that alternative research paradigms—such as adaptive designs, responder analyses, or sequential treatment protocols—may better address the realities of SCI neuromodulation research than traditional placebo-controlled RCTs. While RCTs remain valuable in specific contexts, their limitations in addressing individual variability in complex rehabilitative interventions must be recognized when designing studies and interpreting results.

While sequential within-subject designs mitigate between-person variability, they have limitations [39, 41]. First, generalizability may be reduced if results are driven by subgroup-specific responses. Second, regression to the mean remains a risk when outcomes fluctuate naturally [42]. Third, time-dependent effects—such as health fluctuations, intercurrent infections, or fatigue—can confound interpretation. Mitigation strategies include longer baseline periods to confirm stability, repeated measures across phases, and pre-specifying sensitivity analyses.

## Technical and practical challenges of sham stimulation in tSCS

Difficulties in achieving effective sham stimulation in neuromodulation have previously been identified [9, 10]. The development of valid sham conditions for tSCS presents a distinctive methodological challenge that fundamentally differs from placebo controls in pharmacological or other neuromodulation studies. For a sham condition to be scientifically valid, it must successfully balance two competing requirements: (1) it must be indistinguishable from active treatment from the participant’s perspective, while (2) producing no therapeutic neuromodulatory effects. In the context of tSCS, these requirements create an inherent contradiction that has not been successfully resolved.

While brief stimulation studies (1–2 sessions) with a sham may potentially maintain the illusion of a stimulus [3], sustaining this deception becomes exponentially more challenging across more than 20 sessions spanning two or more months of treatment. Unlike other neuromodulation approaches, such as deep brain stimulation, where patients cannot perceive the stimulation, tSCS produces unmistakable sensations at the site of stimulation that participants quickly learn to recognize and evaluate. The presence or absence of a mild skin reaction at electrode sites, such as redness or warmth under active electrodes, may also influence continued blinding [43].

### The three-fold challenge of valid tSCS sham design

A scientifically robust sham condition for tSCS must simultaneously satisfy three critical requirements:

1. Perceptual equivalence: Generate and maintain a convincing sensory perception that preserves participant belief in receiving active treatment throughout the entire protocol.
2. Physiological inertness: Produces no therapeutic neuromodulatory effects that could impact functional outcomes or confound results.
3. Safety and ethics: Maintain rigorous safety standards and ethical research practices across extended treatment periods.

The Up-LIFT trial experience demonstrated that meeting these requirements simultaneously is very challenging with current technology. Despite extensive development efforts, no tSCS sham paradigm has yet achieved all three criteria. Participants consistently exhibit remarkable sensitivity to stimulation parameters, readily detecting:

- The presence or absence of stimulation.
- Subtle changes in waveform characteristics.
- Current intensity modifications as small as 5 mA.
- Variations in stimulation location.
- Differences in ease of movement due to tSCS facilitation during rehabilitation.

### Minimal stimulation is not inert

Even minimal transcutaneous currents produce detectable effects within the central nervous system [44–46]. This creates a dilemma: currents strong enough to convince participants they are receiving treatment will inevitably produce some neuromodulatory effects. At the same time, genuinely inert stimulation lacks sensory perception and is readily identifiable as sham-treatment by participants.

#### Contextual factors amplifying the sham challenge

The challenge is further compounded by several factors unique to the SCI research context:

- **Knowledge sharing**: Participants frequently interact with each other in clinical settings and through social media platforms, sharing detailed experiences about stimulation sensations and effects.
- **Extended protocols**: The long duration of treatment protocols (24+ sessions) provides ample opportunity for participants to detect inconsistencies or the absence of expected effects.
- **Functional feedback**: Unlike interventions with subtle effects, participants often report immediately improved movement or enhanced muscle contractions during active tSCS.
- **Parameter optimization**: The individualized optimization of stimulation parameters essential for therapeutic efficacy [40] creates distinctive sensory experiences as the amplitude of stimulation is varied to identify the optimal treatment intensity. This is very difficult to replicate in a sham protocol. It would be difficult to mask parameter optimization in a sham as optimization steps are based on changes in response.

Multiple approaches to creating viable sham conditions have been suggested, each with significant limitations that undermine their utility in extended clinical trials (Table 1).

**Table 1:** Attempted Sham Approaches and Their Limitations.

Possible sham stimulation	Details of stimulation	Short-falls
Fade stimulation after 1 min	Gradually reduce the stimulation current to zero after the first minute of stimulation [46]	Participants readily perceive the change or absence of stimulation at the cervical spinal cord level, so the sham is not convincing. The lack of a mild skin reaction may also lead to unblinding [43].
Deliver stimulation to another part of the spinal cord or body	Position electrodes off center or at a point over the spine where the desired treatment effect is not expected	Participants readily perceive the location of stimulation (or it’s absence) over any sensory intact skin, making the sham less convincing. Off-midline stimulation can also activate nerve rootlets and indirectly the spinal cord [44].
Attach electrodes but do not stimulate	No stimulation is delivered, although electrodes and lead wires are placed, and researchers may pretend to set stimulation parameters	Participants will readily perceive the lack of stimulation sensation. Upon discussion with others in the study about what stimulation feels like, they will realize they are in the sham group assignment, resulting in unblinding.
Biphasic stimulation	Less powerful bi-phasic stimulation used as sham, monophasic used for treatment	Bi-phasic stimulation has clear treatment benefits, so the sham is not inert.
Lack of 10 kHz carrier frequency	A 5 or 10 kHz carrier frequency is used for treatment, but not for sham stimulation.	Stimulation over the spinal cord, even without a high carrier frequency waveform, may have detectable effects [45] and functional benefits [63]
Utilize a more surface restricted stimulation modality such as TENS [64, 65]	TENS exhibits limited current penetration to deep structures [64].	A single device may not be able to produce both stimulation types. If two devices are required, this will make blinding of participants and researchers more difficult. The sensory perceptions of stimulation are also likely to differ between TENS and tSCS.

This combination of challenges suggests that traditional sham control approaches may be unsuitable for extended tSCS trials. A new stimulation paradigm would need to be developed to provide convincing sham stimulation. This new sham stimulation should not introduce any additional risks. Therefore, it would need to be tested in smaller preliminary studies to ensure its safety before application in a multi-site clinical trial. Overall, the technical limitations are not merely implementation difficulties but represent a core methodological constraint that necessitates alternative strategies for demonstrating therapeutic efficacy in this field.

## Ethical and practical implications of sham controls in tSCS research

### Ethical tensions in extended sham-controlled trials

Implementing sham controls in lengthy tSCS trials raises significant ethical concerns that extend beyond traditional clinical trial considerations. These challenges must be assessed within the specific context of the vulnerability of the SCI population and the intensive nature of combined neuromodulation-rehabilitation protocols.

The core ethical tension arises from two competing principles:

1. The scientific need for a controlled comparison to establish efficacy.
2. The ethical obligation to minimize the burden and maximize the potential benefit to participants

When participants with SCI commit to months of intensive rehabilitation requiring maximal effort—often with significant hope for functional improvement—the use of sham procedures that provide no therapeutic benefit fundamentally challenges the principle of beneficence. This ethical challenge becomes more pronounced as the duration of the trial increases.

### Trust and motivation as ethical considerations

The extended nature of rehabilitation trials introduces unique concerns about participant trust and motivation that have both ethical and scientific dimensions:

- **Therapeutic relationship integrity:** Repeated deception over months could erode trust in the research team [47], potentially damaging relationships that are central to rehabilitation success.

- **Psychological impact:** Diminished motivation from a suspected sham assignment could directly impact rehabilitation outcomes through lessebo [48] or “nocebo” effects [49].
- **Research team well-being:** Clinicians and therapists often report discomfort with knowingly prolonged deception during intensive rehabilitation relationships [50].

- **Community impact:** Negative experiences can affect willingness to participate in future research, which is particularly important in the close-knit SCI community, where individual experiences widely influence collective attitudes.

These concerns represent practical challenges and fundamental ethical considerations about participant dignity, autonomy, and well-being throughout the research process.

### Participant burden and resource implications

The physical, temporal, and emotional burden on individuals with cervical SCI and their caregivers is substantial when participating in intensive rehabilitation protocols [51]. A comprehensive sham-controlled study design would significantly amplify this burden:

**Table:** 

Protocol Component	Duration	Frequency	Total Visits
**Rehabilitation-only baseline**	**8 weeks**	**3×/week**	**24**
**Randomized sham/treatment**	**8 weeks**	**3×/week**	**24**
**Open-label treatment phase**	**8 weeks**	**3×/week**	**24**
**Total protocol duration**	**24 weeks**		**72 visits**

This design would require:

- A 33% increase in time commitment compared to the Up-LIFT protocol.
- Significantly increased caregiver support requirements.
- Greater transportation and logistical challenges, particularly challenging for participants with tetraplegia.
- Extended exposure to potential health risks, such as viral infections, during study visits.
- Substantial opportunity costs are incurred as participants commit time and energy that could be spent on other rehabilitation approaches.

### Impact on trial feasibility and scientific value

The practical challenges of implementing a traditional sham-controlled design extend beyond participant burden to affect the overall feasibility and scientific validity of the research:

- **Sample size and statistical power:** Without self-controlled comparisons, substantially larger sample sizes would be required to achieve adequate statistical power.
- **Resource constraints:** Significantly increased trial costs could make funding prohibitive, particularly for a condition with relatively low prevalence.
- **Recruitment challenges:** The SCI population is limited, and introducing a known sham arm may further reduce willingness to participate.
- **Attrition risk:** Higher likelihood of participant dropout over extended trial duration, especially if participants suspect sham assignment.
- **Analytical complexity:** Increased dependence on matching quality between tSCS and sham groups introduces additional variables that must be controlled.

Paradoxically, these practical constraints may reduce the scientific value of a traditional RCT approach in this context by introducing selection biases, compromising protocol adherence, and increasing missing data.

### Balancing scientific rigor with practical and ethical realities

These practical and ethical considerations collectively suggest that alternative trial designs may be more feasible and potentially more appropriate for evaluating tSCS interventions when combined with intensive rehabilitation protocols. The scientific ideal of the traditional placebo-controlled RCT must be balanced against the real-world constraints of conducting research with a vulnerable population undergoing intensive, continually optimized long-term interventions.

Alternative approaches that maintain scientific integrity while addressing these concerns include:

- Sequential designs where participants serve as their own controls.
- Adaptive trial designs that allow removal of non-responding cohorts [52].
- Staged protocols that establish proof-of-concept before extended trials.
- Mixed-methods designs that integrate quantitative outcomes with qualitative experiences.

Such approaches may better honor both the scientific obligation to establish treatment efficacy and the ethical responsibility to respect participants’ welfare.

## COVID-19: A critical contextual challenge in trial design

Beyond the inherent technical and ethical challenges already discussed, the Up-LIFT study faced an unprecedented external constraint: it was conducted during the height of the COVID-19 pandemic (January 2021 - June 2022) [6], a period that severely impacted clinical trial execution globally [53], including SCI studies [54]. This timing fundamentally altered the risk-benefit calculus for study design decisions.

### Elevated COVID-19 risks in the SCI population

People with cervical SCI represent a particularly vulnerable population during a respiratory pandemic due to:

- Compromised respiratory function and reduced vital capacity.
- Impaired cough effectiveness and secretion clearance.
- Limited ability to independently perform infection prevention measures.
- Higher rates of comorbidities that compound COVID-19 [55–57].

These factors placed study participants at significantly elevated risk for severe COVID-19 complications and mortality compared to the general population. Therefore, each required in-person study visit represented not merely an inconvenience but a potential life-threatening exposure, particularly before widespread vaccination [58].

### COVID-19 pandemic impact on trial design considerations

The pandemic context critically informed the ethical evaluation of sham controls. While the technical and ethical challenges of sham stimulation existed before COVID-19, the pandemic intensified these concerns:

1. **Amplifying risk-benefit considerations**: The additional visits required to offer an equal amount of delayed treatment following sham stimulation would have increased COVID-19 exposure by approximately 33% for participants randomized to the sham arm of the study.
2. **Introducing continuity concerns**: Pandemic-related research shutdowns created substantial uncertainty about protocol completion, with longer protocols facing a higher risk of interruption [59].
3. **Complicating transportation logistics**: Pandemic restrictions and safety precautions made the already challenging transportation requirements for participants with tetraplegia even more difficult.
4. **Reducing available support**: Limitations on caregivers and support personnel allowed to accompany them to in-clinic research visits further strained participant resources.

### The pandemic as a decision accelerator

Rather than creating entirely new challenges, the pandemic accelerated and intensified the existing technical and ethical tensions in trial design. This included the confluence of:

- Technical challenge of creating a truly effective sham (as detailed in section 4).
- Ethical concerns about participant burden (discussed in the Section 5: Ethical Implications section).
- Additional pandemic-related risks for an already vulnerable population.
- Strong regulatory recommendations to implement risk reduction measures.

This combination reinforced the conclusion that alternative trial designs were necessary, especially considering the time-sensitive need to deliver potential therapeutic benefits to the SCI community amid a period of heightened health vulnerability of uncertain duration.

Looking forward, lessons from the pandemic support the value of hybrid and decentralized trial approaches. Remote monitoring, gamified task practice, and real-time virtual therapist supervision could reduce participant risk and burden while preserving data quality in future studies [54].

The pandemic context thus provides a clear example of how clinical trial design must sometimes adapt to external realities while maintaining scientific integrity. The Up-LIFT study design represented a reasoned response to these extraordinary circumstances, balancing scientific rigor with participant protection in an unprecedented global health crisis [51].

## Alternative design approaches for tSCS clinical research

**Fig. 2:**
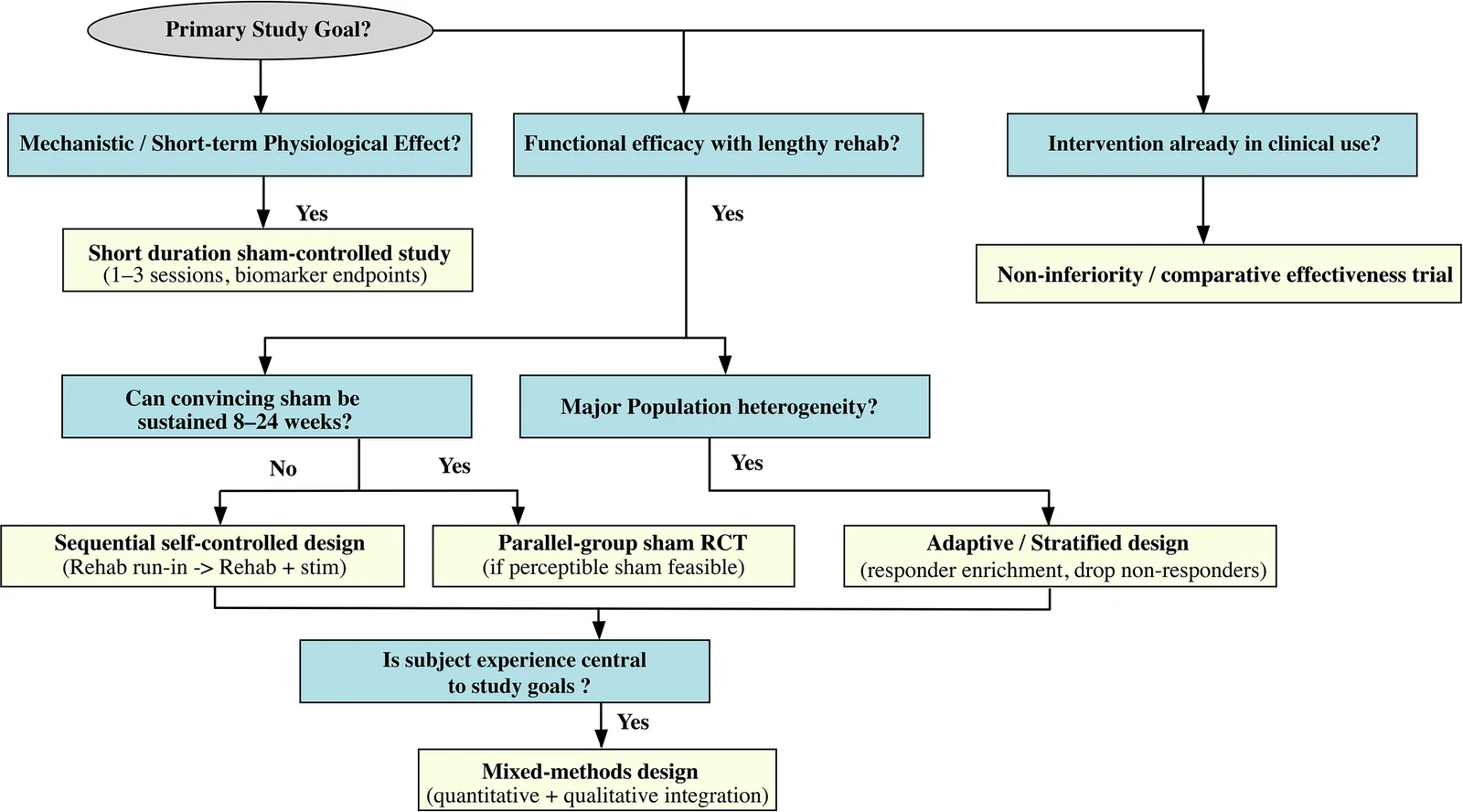
Decision tree for selecting trial designs in SCI neuromodulation research. Flowchart summarizing considerations for choosing among trial designs when evaluating transcutaneous spinal cord stimulation (tSCS) or related neuromodulation interventions. Short-duration sham-controlled studies are best suited for mechanistic or biomarker-driven investigations, while longer functional efficacy trials require evaluation of whether sham conditions can be maintained. If not feasible, sequential self-controlled designs may be used; if feasible, parallel-group sham-controlled RCTs are appropriate. In the presence of major population heterogeneity, adaptive or stratified approaches may improve efficiency. When interventions are already in clinical use, non-inferiority or comparative effectiveness trials may be most informative. Finally, mixed-methods designs allow integration of quantitative and qualitative outcomes when patient experience is central to the study goals.

Given the challenges with traditional sham-controlled designs in extended tSCS trials, several alternative methodological approaches warrant consideration (Fig. 2):

### Sequential self-controlled designs

As implemented in the Up-LIFT trial, having participants serve as their own controls through a rehabilitation-only phase followed by a rehabilitation combined with stimulation phase offers the advantages of controlling for individual variability and ensuring that all participants receive potential benefits. One limitation is that it cannot fully account for time-dependent effects that are independent of the intervention.

### Short-duration proof-of-concept studies

Brief protocols (1-2 sessions) can effectively utilize sham conditions to establish neurophysiological effects through biomarkers before advancing to extended therapeutic trials. This approach maintains blinding integrity while minimizing participant burden [3].

### Biomarker-guided approaches

Neurophysiological measurements and functional biomarkers can provide objective evidence of stimulation effects that are independent of functional outcomes, thereby supporting mechanistic understanding and efficacy evaluation. Neurophysiological measurements can serve as objective outcomes. Candidate biomarkers include changes in EMG and recruitment curves [60], MEP amplitude [3], H-reflex modulation [61], and EEG measures of sensorimotor engagement [62]. These measures are less susceptible to placebo effects than functional outcomes. An important advantage is that they provide mechanism-based evidence of stimulation effects. Reliability is, however, a challenge; session-to-session variability can be substantial, underscoring the need for normalization strategies and careful interpretation of their correlation with functional outcomes. The correspondence between biomarkers and functional improvements is not always direct.

### Combination therapy approaches

Pairing tSCS with pharmacological interventions can create study arms in which all participants receive some active treatment [32], maintaining motivation while allowing comparison of different therapeutic mechanisms.

### Non-inferiority and comparative effectiveness trials

As tSCS enters clinical practice, comparing new stimulation protocols or devices against established interventions can advance the field while ensuring all participants receive active treatment.

### Adaptive trial designs for SCI heterogeneity

Trials that utilize stratification methods and sequentially refine the responder and non-responder populations and contributing features [52]. The Up-LIFT study identified some responder criteria in post-hoc analyses that can inform new studies [6].

These alternative designs represent a pragmatic response to the unique challenges of neuromodulation research in rehabilitation contexts. They potentially offer ethically and scientifically sound alternative approaches beyond traditional RCTs for such interventions.

## Summary and future directions

The Up-LIFT trial navigated complex methodological issues to generate valuable evidence supporting the efficacy of tSCS in improving upper extremity function in chronic tetraplegia [6]. This ultimately contributed to the FDA’s approval of this intervention. This achievement demonstrates that innovative trial designs and endpoints can successfully balance scientific rigor with practical and ethical considerations.

Future studies are expected to further refine tSCS applications through:

- Optimization of stimulation parameters for different injury populations.
- Development of combination approaches with other therapeutic strategies.
- Investigation of home-based application to reduce participation burden.
- Exploration of novel outcome measures and biomarkers to quantify effects.

While developing and validating improved sham paradigms remains important for advancing the field, researchers must continue to prioritize designs that:

1. Respect the significant commitment required from participants with SCI.
2. Acknowledge the unique challenges of extended rehabilitation protocols.
3. Maximize the potential for therapeutic benefit while minimizing burden.
4. Generate robust evidence that supports clinical implementation.
5. No clinical trial design is perfect, and the pursuit of methodological ideals must be balanced with the practical realities of advancing therapeutic options for people with SCI. Successful clinical trials are crucial not only for regulatory approval but also for fostering investment and building confidence in the SCI field.

## Conclusion

Three critical constraints justified the UP-LIFT study design: (1) the absence of a safe, inert, and convincing sham stimulation paradigm suitable for extended protocols; (2) the substantial additional burden that sham control would place on participants with SCI; and (3) the heightened risks associated with increased study visits during the COVID-19 pandemic.

These constraints necessitated approaches to clinical trial design that balanced scientific rigor with practical feasibility and ethical considerations. The resulting trial navigated these challenges successfully and generated evidence robust enough to support regulatory approval, demonstrating that methodological innovation can advance clinical care even when traditional RCT approaches are unsuitable.

Future research should pursue dual paths: developing improved sham paradigms while continuing to advance alternative trial methodologies that can robustly demonstrate efficacy without imposing undue burden on vulnerable populations. This balanced approach will best serve both the scientific advancement of the field and the ultimate goal of translating promising interventions into meaningful improvements in function and quality of life for people living with spinal cord injury.

## Data Availability

The data referred to for the Up-LIFT study is attached as a supplement in Moritz, C., Field-Fote, E.C., Tefertiller, C. et al. Non-invasive spinal cord electrical stimulation for arm and hand function in chronic tetraplegia: a safety and efficacy trial. *Nat Med* **30**, 1276–1283 (2024). https://doi.org/10.1038/s41591-024-02940-9. Supplementary Data 1. That study includes the study ethics and approval, and consent to participate and employed the ASIA/ISCoS International standards for Neurological Classification of Spinal Cord Injury (ISNCSCI).
